# Double-stapled anastomosis without “dog-ears” reduces the anastomotic leakage in laparoscopic anterior resection of rectal cancer: A prospective, randomized, controlled study

**DOI:** 10.3389/fsurg.2022.1003854

**Published:** 2023-01-06

**Authors:** Yuanfeng Yang, Feng Ding, Tianbao Xu, Zhen Pan, Jinfu Zhuang, Xing Liu, Guoxian Guan

**Affiliations:** ^1^ Department of Colorectal Surgery, The First Affiliated Hospital of Fujian Medical University, Fuzhou, China; ^2^ Department of Colorectal Surgery, Fujian Medical University Union Hospital, Fuzhou, China

**Keywords:** middle and high rectal cancer, anastomotic leakage, modified double-stapling technique, laparoscopic, anterior resection

## Abstract

**Background:**

Anastomotic leakage (AL) is a major cause of postoperative morbidity and mortality in the treatment of colorectal cancer. The aim of this study was to investigate whether the resection of “dog-ears” in laparoscopic anterior resection of rectal cancer (called modified double-stapling technique, MDST) could reduce the rate of AL in patients with middle and high rectal cancer, as compared with the conventional double-stapling technique (DST).

**Methods:**

The clinical data of 232 patients with middle and high rectal cancer were prospectively collected from September 2015 to October 2018. They were randomly divided into the MDST group (*n* = 116) and the DST group (*n* = 116) and the data were prospectively analyzed. Morbidity and AL rate were compared between the two groups.

**Results:**

Patient demographics, tumor size, and time of first flatus were similar between the two groups. No difference was observed in the operation time between the two groups. The AL rate was significantly lower in the MDST group than in the DST group (3.4 vs. 11.2%, *p *= 0.032). The age and anastomotic technique were the factors associated with AL according to the multivariate analysis. The location of the AL in the DST group was further investigated, revealing that AL was in the same place as the “dog-ears” (11/13, 84.6%).

**Conclusions:**

Our prospective comparative study demonstrated that MDST have a better short-term outcome in reducing AL compared with DST. Therefore, this technique could be an alternative approach to maximize the benefit of laparoscopic anterior resection on patients with middle and high rectal cancer. The “dog-ears” create stapled corners potentially ischemic, since they represent the area with high incidence of AL.

(NCT:02770911)

## Introduction

The mortality and morbidity due to colorectal cancer is increasing worldwide. Rectal cancer in China represents approximately two-third of all cases of colorectal cancer (1). The main treatment for rectal cancer is surgery, such as anterior resection. Several results from randomized clinical trials on rectal cancer patients demonstrated that laparoscopic surgery is consistent with open surgery regarding the long-term local recurrence, disease-free survival, and overall survival (2–5). Laparoscopic surgery for rectal cancer is less invasive and facilitate the anastomosis in the deep pelvis compared with open surgery (4–7). However, many large-scale randomized controlled trials (RCTs) compared the rate of postoperative complication between laparoscopic and open surgery, discovering no reduced the rate of anastomotic leakage (AL) (4–7).

AL in rectal cancer surgery is one of the most critical complications affecting not only the short-term outcomes but also the long-term (8–11). Moreover, recent studies revealed that the incidence of AL is from 6% to 17% after the anterior resection of the rectal cancer by laparoscopic surgery (12–14). Therefore, it is not yet possible to reduce the incidence of AL after rectal surgery. Double-stapling technique (DST) is the key step representing the technical difficulty when performing laparoscopic anterior resection for rectal cancer (15), and it is the major contributing factor of AL. DST is characterized by the placement of at least two staple lines crossing each other, creating stapled corners (called “dog-ears”) potentially ischemic, which represent the area with high incidence of AL (16).

DST for low anterior resection, reported first by Knight et al. in 1980, involves rectal transection with a linear stapler and the creation of anastomosis with a circular stapler (17). Kang et al. (18) reported a modified double-stapling technique (MDST) to remove the dog-ears in open surgery by excising the distal bowel. Their results showed that the AL rate was remarkably reduced in MDST, but their procedure was successful on sigmoid colon cancer, that was powerless for middle and high rectal cancer. Our previous study involved 110 patients with high rectal cancer and sigmoid colon cancer subjected to surgery performed entirely by MDST (19). Our previous results preliminary confirmed that MDST reduces the AL rate after laparoscopic anterior resection compared to what it was demonstrated in other previous results.

Therefore, the present study was a prospective and randomized comparison that investigates the feasibility and impact on surgical outcomes of MDST compared with DST in laparoscopic anterior resection performed on patients with middle and high rectal cancer.

## Materials and methods

### Patient demographics

**Figure 1:**
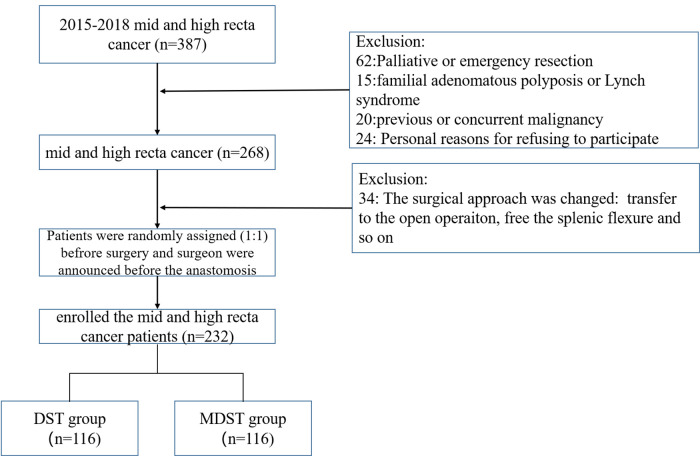
The patients flow chart.

The clinical data of 232 patients with middle and high rectal cancer who underwent laparoscopic anterior resection were prospectively collected between September 2015 and October 2018. Patients were randomly divided into MDST group (*n* = 116) and DST group (*n* = 116). The inclusion criteria where the following: age between 18 and 80 years, middle and high rectal cancer with the lower margin of the tumor located above the retroperitoneal fold. The exclusion criteria were as follows: patients who underwent neoadjuvant or adjuvant chemoradiotherapy, patients with an American Society of Anesthesiologists score IV, patients who had distant metastasis at initial diagnosis. All patients gave the written informed consent, and all the procedures were performed by the same team of board-certified colorectal surgeons at the Colorectal Surgery Department of Fujian Medical University, Union Hospital. The study was approved by the hospital review board (2016YF012-01). The patients flow chart is shown in Figure 1.

### Randomization and masking

Patients were recruited at the participating hospital before the start of any treatment and randomly divided (1:1) by ProMISe data management system (version 4.0) using a stratified and randomly varying block design applied to both the experimental group (MDST) and the standard group (DST). The stratifying factors were institution, ECOG scale of performance status (0 or 1), cT stage (cT2–cT3 or cT4), and cN stage (cN– or cN+). Randomization was coordinated by the Clinical Research Center. The investigators were blinded to the treatment assignment during the evaluation of the primary endpoint until the prespecified number of events was reached. However, patients and clinical staff were not blinded to the assigned group due to the nature of the intervention.

### MDST in laparoscopic anterior resection

**Figure 2:**
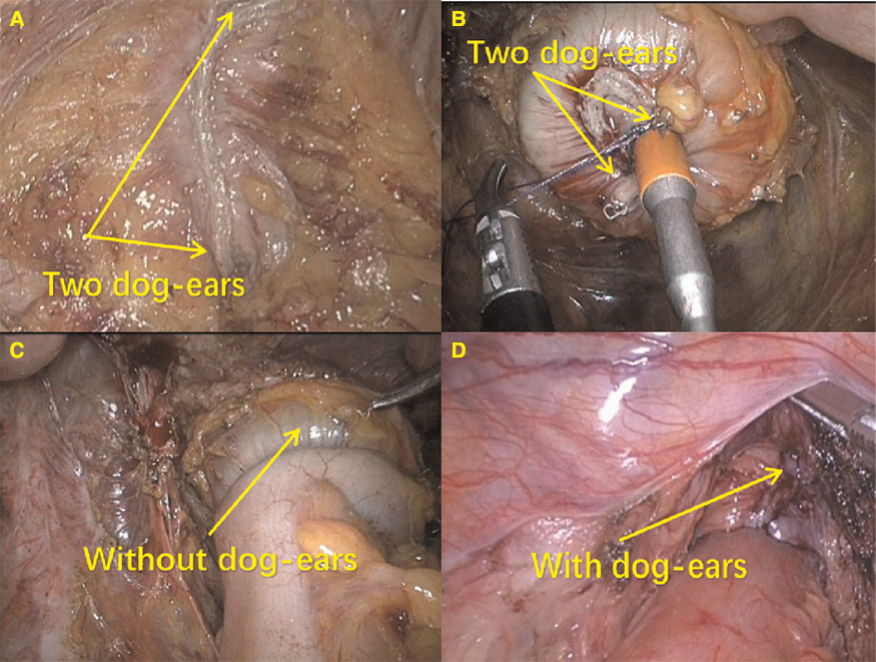
End-to-end anastomosis by DST and MDST. (**A**) Suturing two corners of the staple line around the trocar; (**B**) Without “dog ears” after anastomosis; (**C**) Distal rectum with “dog ears”; (**D**) With “dog ears” after anastomosis.

Our technique of laparoscopic surgery for the treatment of the left-sided colorectal cancer was previously described in detail (20, 21). Patient preparation and the dissection method before anastomosis were not different between the MDST group and the DST group. The mesorectum at the point where the rectal wall was transected, was mobilized for a clean colorectal anastomosis before the application of the laparoscopic linear stapler (ECR60G, Ethicon Endo-Surgery Inc.) to the distal bowel, and the surgeon kept the layer of the longitudinal muscle intact. The transection of the distal rectum was performed using one cartridge. The assistant inserted the circular stapler device (CDH33, Ethicon Endo-Surgery Inc.) through the anus up to the close rectal lumen after the removal of the anvil and the formation of the staple line forming the dog-ears, as clearly shown on the rectal stump. Then the trocar was extended, and the rectal wall was perforated by gently rotating the instrument. The surgeon made a laparoscopic suture on the two dog-ears in the patients of the MDST group using 3-0 monofilament sutures, then the trocar was fully extended, and the dog-ears were removed from the staple line around the trocar by means of a suture tied through them (Figure 2A). In this way, the staple line was kept within the circular knife when the circular stapler was closed. Then a true end-to-end anastomosis was performed after stapler firing (Figures 2B,C). However, the surgeon did not make a laparoscopic suture on the two dog-ears in the patients of the DST group (Figure 2D). After anastomosis, the air leak test was performed in the patients of both groups. A drain was routinely placed in the pelvic cavity, near the anastomosis, before the closure of the abdominal incision. No protective stoma was made.

### Perioperative outcomes

Perioperative outcomes including duration of the operation, length of the resection margin, time to first flatus, resumption of a soft diet, length of hospital stay, and morbidity were compared between the two groups. AL was diagnosed based on clinical signs or image studies. The clinical signs included abdominal pain or fever, production of pus or feces through the indwelling drain, and local or generalized peritonitis. Radiologic evaluation was performed to confirm the clinical suspicion.

### Statistical analysis

Statistical analysis was performed using SPSS (version 22; IBM, Armonk, NY).

The *χ*^2^ test or Fisher's exact test was used to compare the categorical variables, while Student's *t*-test or Mann–Whitney *U* test was used to compare continuous variables. A *p* value less than 0.05 was considered statistically significant.

## Results

### Patient characteristics

A total of 387 middle and high rectal cancer patients accepted to be subjected to the anterior resection by our surgery team. In addition, distant metastases were found in 50 patients and 12 patients accepted the emergency resection. Fifteen patients suffered from familial adenomatous polyposis or Lynch syndrome, 20 patients had previous or concurrent malignancies, and 24 patients refused to participate to the clinical trial. A total of the 268 patients were enrolled in the clinical trial. However, during the surgery 12 patients were transferred to open surgery and 22 patients accepted the left hemi-colectomy. The above patients were excluded from the clinical trial. Finally, 232 patients were randomly assigned (1:1) to the DST and MDST group. Age, gender, previous history of abdominal surgery, body mass index, American Society of Anesthesiologists grade, and tumor location in patients who underwent MDST were similar to those who underwent DST. No difference was found on pathologic outcomes such as tumor size, number of metastatic lymph nodes and pathological TNM stage between the two groups (Table 1).

**Table 1:** Comparison of patient characteristics and pathologic outcomes.

Variable	MDST (*N* = 116)	DST (*N* = 116)	*p* value
Age (years)	63.0 ± 8.5	61.3 ± 9.3	0.148
Gender			0.146
Male	70 (60.3)	57 (49.1)	
Female	46 (39.7)	59 (50.9)	
BMI (kg/m2)	22.8 ± 2.9	22.5 ± 2.9	0.448
Smoking history	47 (40.5)	38 (32.8)	0.220
PAS	21 (18.1)	23 (19.8)	0.738
ASA grade			0.836
I	94 (81.0)	94 (81.0)	
II	21 (18.1)	20 (17.2)	
III	1 (0.9)	2 (1.7)	
CEA (ng/ml)			0.890
≤5.0	76 (65.5)	75 (64.7)	
>5.0	40 (34.5)	41 (35.3)	
CA199 (U/ml)			0.819
≤37.0	105 (90.5)	106 (91.4)	
>37.0	11 (9.5)	10 (8.6)	
Tumor location (cm)	11.6 ± 3.2	11.5 ± 3.2	0.742
Tumor size (cm)	3.8 ± 1.3	3.9 ± 1.4	0.390
Length of DRM (cm)	2.4 ± 1.4	2.4 ± 1.2	0.992
No.of metastatic LNs	1.6 ± 3.0	1.7 ± 3.4	0.853
Nerve invasion	22 (19.0)	21 (18.1)	0.890
Vascular tumors bolt	20 (17.2)	21 (18.1)	0.863
Pathological stage			0.864
I	25 (21.6)	26 (22.4)	
II	41 (35.3)	44 (37.9)	
III	50 (43.1)	46 (39.7)	

BMI, body mass index; PAS, previous abdominal surgery; ASA, American Society of Anesthesiologists; CEA, carcinoembryonic antigen; CA199, Carbohydrate antigen199; DRM, distal resection margin; LN, lymph node; MDST, modified double-stapling technique; DST, double-stapling technique.

### Perioperative outcomes

The perioperative outcomes and postoperative recovery were the important results to evaluate the anastomotic techniques. In the present study, the perioperative outcomes and postoperative recovery of each group were compared. The result demonstrated that the operation time and blood loss did not increase in the MDST group (*p *= 0.581 and *p *= 0.209). Moreover, the result of the postoperative complication revealed that the total rate of complication between the two groups was similar (DST, 12.9% vs. MDST, 6.9%; *p *= 0.124). However, the AL rate was significantly lower in the MDST group compared with the DST group (DST, 11.2% vs. MDST, 3.4%; *p *= 0.032). Other postoperative complications such as anastomotic hemorrhage, ileus, wound infection, chyle leakage and urinary tract infection were not different between the two groups (all *p *> 0.05). The result of the postoperative recovery demonstrated that the length of hospital stay and the time of first bowel movement in the patients of the MDST group were significantly decreased (*p *= 0.032 and *p *= 0.005, respectively). No difference in the time of first flatus, time of resuming a soft diet, and time of resuming a semi-liquid diet was found between the two groups (Table 2).

**Table 2:** Comparison of perioperative outcomes and postoperative recovery.

Variable	MDST (*N* = 116)	DST (*N* = 116)	*p* value
Operation time (min)	126 (114–145)	128 (115–150)	0.581
Blood loss (ml)	30 (20–50)	30 (20–50)	0.209
Anal drainage tube	32 (27.6)	28 (24.1)	0.549
Postoperative complications	8 (6.9)	15 (12.9)	0.124
Anastomotic leakage	4 (3.4)	13 (11.2)	**0**.**032**
Anastomotic hemorrhage	3 (2.6)	1 (0.9)	0.620
Ileus	0 (0.0)	1 (0.9)	1.000
Wound infection	0 (0.0)	2 (1.7)	0.156
Chyle leakage	1 (0.9)	3 (2.6)	0.313
Urinary tract infection	0 (0.0)	2 (1.7)	0.156
Re-exploration	1 (0.9)	2 (1.7)	0.561
Recovery			
First flatus movement (hours)	19.0 (17.0–21.8)	19.7 (17.4–24.9)	0.185
First bowel movement (hours)	47.3 (43.9–52.4)	50.5 (45.7–59.1)	**0**.**005**
Soft diet resumed (hours)	17.4 (15.9–20.3)	18.1 (16.2–20.6)	0.224
Semi-liquid diet resumed (hours)	66.8 (64.6–69.2)	66.7 (63.4–67.0)	0.466
Length of hospital stay (days)	6.7 ± 1.6	7.7 ± 4.7	**0**.**032**

MDST, modified double-stapling technique; DST, double-stapling technique.

### Factors associated with AL

AL in rectal cancer surgery is one of the most critical complications, and it affects not only short-term outcomes but also the long-term ones such as local recurrence and overall survival (8–11). The risk factors for the AL were evaluated, and the univariate analysis demonstrated that age (*p *= 0.014) and MDST (*p *= 0.032) were the protective factors for AL. The multivariate analysis, revealed that younger (OR = 0.945, CI: 0.900–0.993, *p *= 0.024) and MDST (OR = 0.308, CI: 0.096–0.987, *p *= 0.047) were the protect factors associated with AL (Table 3).

**Table 3:** Factors associated with anastomotic leakage.

Variable	Univariate analysis	*p* value	Multivariate analysis	*p* value
	OR value (95%CI)		OR value (95%CI)	
Age	**0.941** **(0.897–0.988)**	**0**.**014**	**0.945** **(0.900–0.993)**	**0**.**024**
Gender	1.152 (0.423–3.141)	0.281		
BMI	1.040 (0.877–1.233)	0.655		
Smoking history	0.486 (0.180–1.311)	0.154		
Anastomotic technique	**0.283** **(0.089–0.896)**	**0**.**032**	**0.308** **(0.096–0.987)**	**0**.**047**
Operation time	1.012 (0.996–1.028)	0.147		
Blood loss	1.005 (0.990–1.021)	0.492		
Length of DRM	0.971 (0.666–1.416)	0.878		
Tumor size	1.221 (0.869–1.717)	0.250		
PAS	1.100 (0.302–4.005)	0.886		
Tumor location	0.924 (0.785–1.087)	0.339		
ASA grade	1.570 (0.586–4.209)	0.370		
pT	0.980 (0.532–1.803)	0.947		
pN	0.413 (0.161–1.059)	0.066		

BMI, body mass index; DRM, distal resection margin; PAS, previous abdominal surgery; ASA, American Society of Anesthesiologists; OR, odd ratio; CI, confidence intervals.

**Figure 3:**
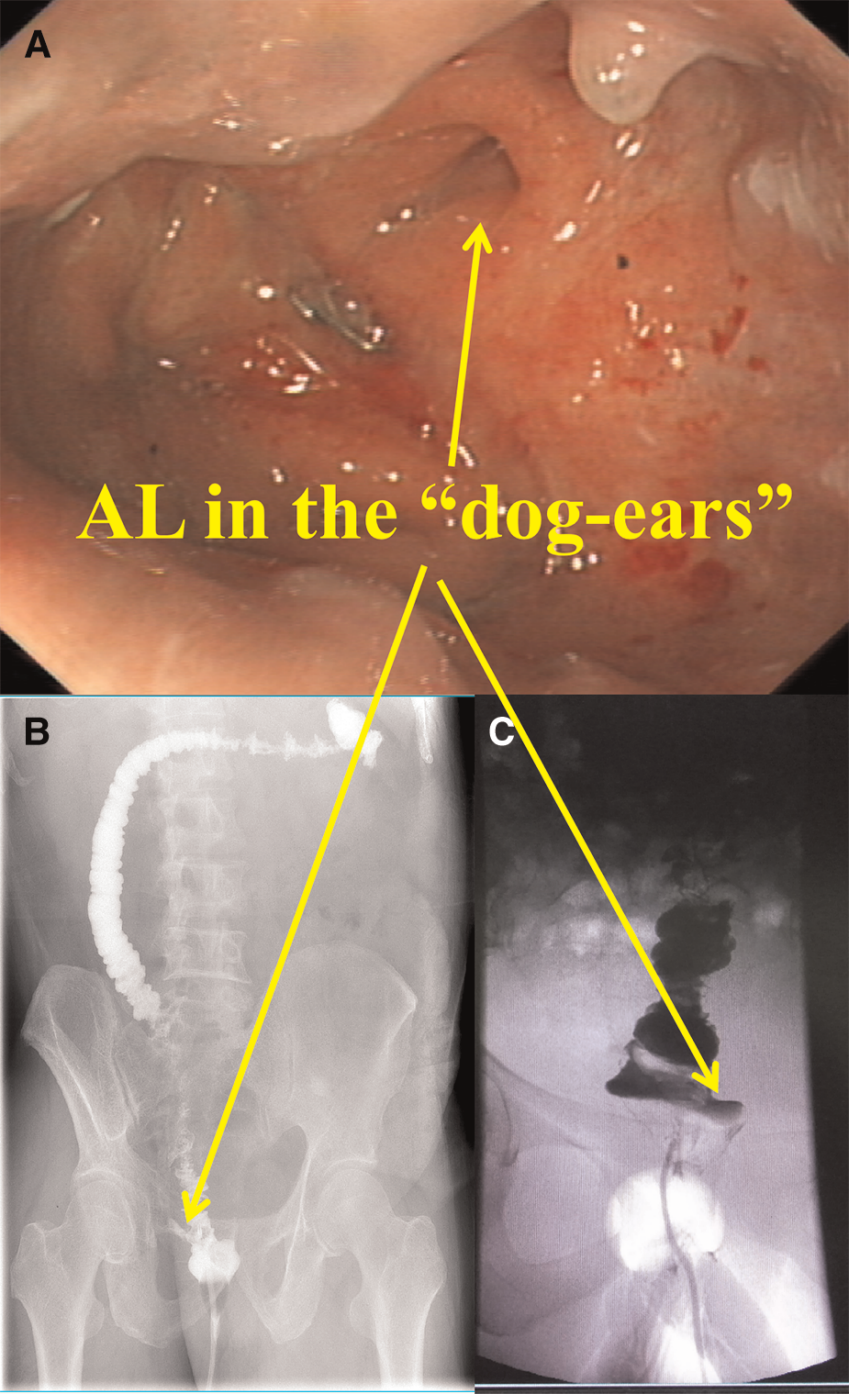
Diagram of AL. (**A**) The colonoscopy of the AL patinets. (**B**) and (**C**) The x-ray of the AL patinets.

The patients with AL in the DST group were subjected to colonoscopy one week after the surgery to further confirm the location of AL in the DST group (Table 4 and Figure 3). AL was located were the “dog-ears” were located in the DST groups (11/13, 84.6%). The above result revealed that the “dog-ears” as the two staple lines crossing each other, created potentially ischemic stapled corners with high incidence of AL (16).

**Table 4:** Clinicopathological details of 13 AL patients in DST group.

No	Age (yrs)	Sex	BMI	Pathology stage	The size of leakage	Contain dog-ear	Stay after surgery (days)	Re-exploration	AL level
1	54	F	29.4	T3N1M0	1/4	Yes	11	No	B
2	49	M	18.7	T2N1M0	1/4	Yes	34	Yes	C
3	43	M	19.9	T3N0M0	1/5	Yes	8	No	B
4	58	F	23.0	T3N0M0	1/6	Yes	8	No	B
5	69	F	23.5	T3N0M0	1/5	Yes	10	No	B
6	63	F	23.6	T3N0M0	1/5	Yes	14	No	A
7	55	M	27.0	T3N0M0	1/6	No	21	No	A
8	67	F	19.0	T3N0M0	1/4	Yes	20	Yes	C
9	61	F	27.1	T3N1M0	1/12	Yes	20	No	A
10	54	F	25.8	T2N0M0	1/12	No	29	No	A
11	70	M	23.0	T3N1M0	1/6	Yes	28	No	B
12	64	M	18.4	T3N0M0	1/5	Yes	17	No	A
13	48	F	24.8	T1N0M0	1/6	Yes	11	No	B

M, male; F, female; AL, anastomotic leakage.

## Discussion

In recent years, laparoscopic rectal surgery has become popular because of the its minimally invasiveness and advantage of its magnified view (2–5). This surgery requires a safe and precise technique for completing the total mesorectal excision within the narrow pelvis. However, many large-scale RCTs comparing the oncological outcomes and long-term outcomes between laparoscopic and open surgery did not reveal a reduced postoperative complication rate, including AL (2–5). The present study demonstrated that MDST could reduce the incidence of AL in the laparoscopic low anterior resection (22–26). Moreover, the location of AL was explored in the DST group, and the result demonstrated that it was located in the same place of the “dog-ears”. The above result indicated that the removal of the “dog-ears” could efficiently decrease AL in the laparoscopic high/mid anterior resection (22–26).

DST for low anterior resection was firstly reported by Knight et al., in 1980, and involves rectal transection with a linear stapler and the creation of an anastomosis with a circular stapler. This method commonly used in the past to perform open surgery contributed to the recent explosive spread of laparoscopic low anterior resection (17). Despite various studies explored the risk factors for AL in the open low anterior resection, few reports explored them in laparoscopic low anterior resection (22–26). The low anterior resection is often performed with a stoma creation to reduce AL or reoperation (15, 27–30). However, since stoma can cause stoma-related complications and cosmetic problems, it should not be performed in all patients with low anterior resection. Thus, to explore the risk factors for AL in patients without stoma can help surgeons to decide to create stoma or not.

The anastomosis is the key of the success and difficulty of the whole surgery, that is directly related to the postoperative functional recovery and complications of patients with middle and high rectal cancer (15, 17). Conventional DST significantly improved the anal preservation rate of rectal cancer patients, but the incidence of AL was not significantly improved (31), The occurrence of AL might be closely related to the following points, except for the anastomotic blood supply and anastomotic tension (32). First, when the distal rectum with linear cutting is closer to the transverse cutting, it gradually tapered off until becoming very thin. “Dog-ears” were produced on both sides when the two ends were closed using tubular anastomotic matches. The tissue of the dog-ear is relatively weak due to the elongation and thinning. This could be the anatomical and histological basis of AL and diverticulitis. Second, a “T” shaped junction of the suture line was formed after anastomosis, which is a recessive ischemia area and high-risk area of AL. Many scientists noticed its dangerous consequences and called it the “dangerous triangle” (21). Finally, at present a consensus was reached regarding the inevitable risk of anastomotic leakage after pre-rectal resection. The AL rate has been reported by various studies as very different. Thus, further studies are needed to establish a relationship with the surgical design and operation.

Previous studies reported that dog-ears retained by the traditional DST are an important risk factor for AL (33, 34). In addition, the anastomotic stenosis rate of traditional DST with retained “dog-ears” were higher than that in the single stapling technique (35). Many surgeons made efforts in removing the dog-ears in order to reduce AL. Marecik et al. (36). described a reliable single-stapled double purse-string anastomosis, which could also remove the dog-ears. Only one case of class C AL occurred in this study (0.6%). Sileri et al. (37). used the KOLTM circular stapler for the removal of the dog-ears. They used two straight and long needles and four 2-0 prolene sutures to pull the staple line on the rectal stump into the rectal lumen through the circular anal dilator placed in the anus. Kim et al. (21) reported that the single stapled technique without dog-ears could be intracorporeally performed with the application of robotic surgery. The AL rate was not significantly different between the single stapled technique group and DST group in this study. Kang et al. (32) reported a modified MDST to eliminate the dog-ears in open surgery by pulling out the distal bowel. The AL rate was clearly reduced in MDST. Kang's procedure was not performed entirely using laparoscopy, and the procedure was mainly studied on sigmoid colon cancer, but it was not effective when applied to middle and high rectal cancer.

At present, many reports are available regarding the use of DST for rectal cancer, but only few reports are available on the radical cure, safety and short-term efficacy of DST and MDST. In this study, these two surgical techniques were compared, with the aim of providing an individualized treatment and more reasonable surgical strategies for patients with middle and high rectal cancer. A prospective randomized controlled study was performed, and the distal rectum insufflate was removed using Johnson EC60 linear cutting anastomat, which is 5 cm above the anal edge. The two horns of the distal intestinal canal were dragged into the Johnson GF-33 mm tubular anastomat nail “warehouse” within “end-to-end” anastomosis with stitches during the laparoscopic surgery. No “dog-ears” and “dangerous triangle” remained after the anastomosis as long as the two-cut end anastomosis ring was complete, all without prophylactic enterostomy. As regard the rectal stump, which is less than 5 cm below the anal margin, it is difficult to suture and embed the two “dog-ears” under the laparoscope, while it is easy to stretch and split the rectal stump with the Johnson GF-33 mm tubular staplers. Therefore, this study only discussed the application of this method for middle and high rectal cancer, which could significantly reduce AL rate and anterior resection syndrome without increasing the difficulty and cost of the surgery. In addition, it avoids the pain caused by prophylactic enterostomy and produces economic and social benefits.

Several limitations of our study should be mentioned. First, the present randomized controlled trial was performed on patients from one center. Thus, the results obtained should be verified by a multi-center randomized controlled trial involving different countries and races. Second, a higher number of patients was not used in the present study due to the limitation of the study time. Thirdly, we did not explore the rate od th AL in the locally advanced rectal patients who accepted the neo-chemoradiotherapy, which has the high rates of the AL. Despite these limitations, our study increases our understanding of the impact of MDST in the low anterior resection.

This study revealed that the AL rate in the MDST patients was lower than that in the DST patients, and the postoperative defecation time was reduced. Thus, MDST was the protective factor for AL in the laparoscopic low anterior resection. Moreover, the location of the leakage was associated with the residue “dog-ears”.

## Data Availability

The original contributions presented in the study are included in the article/Supplementary Material, further inquiries can be directed to the corresponding author/s.
